# Supplementary material for: Rising dengue risk with increasing El Niño–Southern Oscillation amplitude and teleconnections
Source: Nat Commun. 2025 Sep 29;16:8629. doi: 10.1038/s41467-025-63655-0 (PMC12480917; doi:10.1038/s41467-025-63655-0)
Supplement: Supplementary file 1 — Supplementary Information [file 41467_2025_63655_MOESM1_ESM.pdf]

Supplementary Information

This supplementary material contains the following Supplementary Figures, Tables, and Text:

Supplementary Figures:

Supplementary Figs. 1–14

Supplementary Tables:

Supplementary Tables 1–7

Supplementary Text:

Methods to estimate annual case counts for 2024 accounting for reporting delays and yet-to-be-reported data

Supplementary Figures

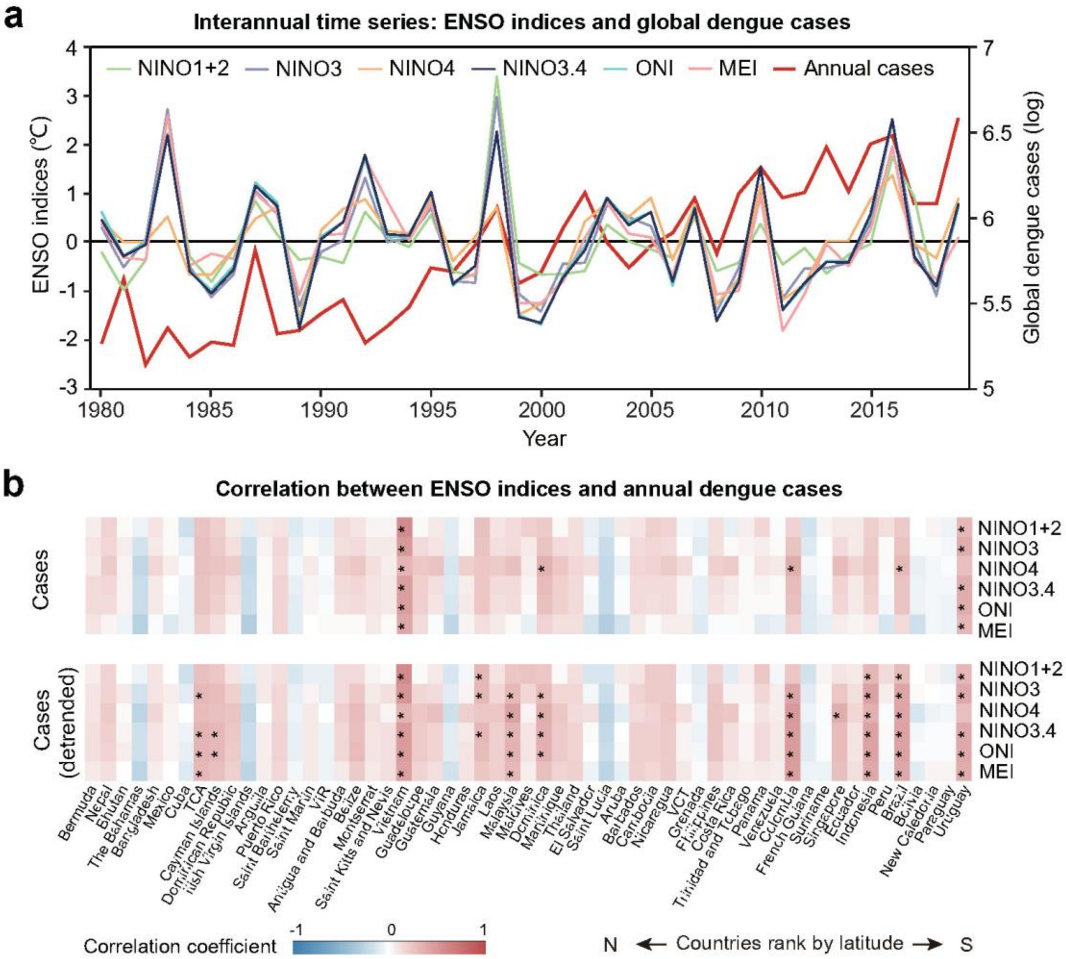

Supplementary Fig. 1 Association between ENSO indices and dengue epidemics in 1980–2019. a, Interannual time series of DJF ENSO indices (blue lines) and globally reported dengue cases (red line). **b**, Pearson correlation between detrended/non-detrended DJF ENSO indices and annual dengue cases in 57 tropical countries. The correlation coefficient ranges from negative values (blue) to positive values (red). Significant correlations ($r > 0.304$, $P \leq 0.05$) are denoted by an asterisk. TCA: Turks and Caicos Islands; VIR: United States Virgin Islands; VCT: Saint Vincent and the Grenadines. Source data are provided as a Source Data file.

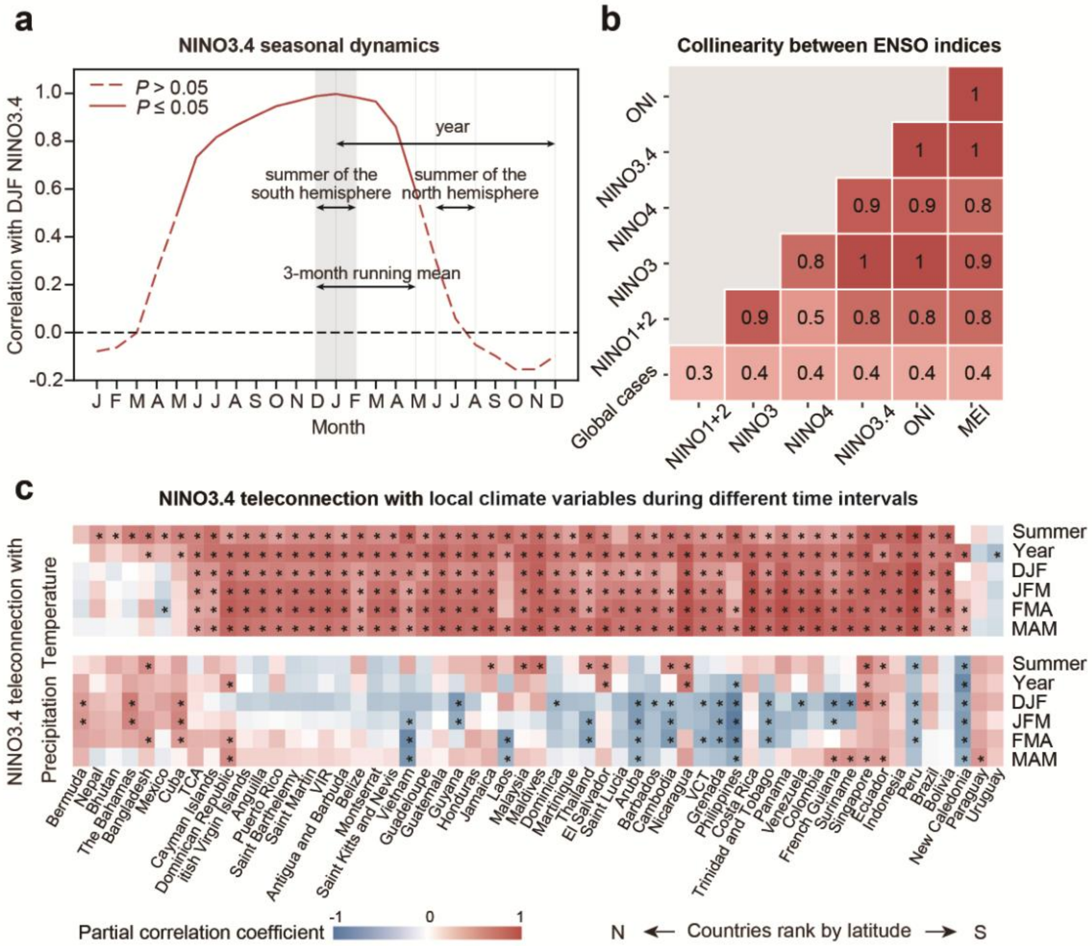

Supplementary Fig. 2 ENSO indices and teleconnections with local climate. a, Seasonal dynamics of the NINO3.4 index and different time intervals of teleconnections. The solid line denotes significant correlation coefficients ($P \leq 0.05$) and the dashed line denotes insignificant correlation coefficients ($P > 0.05$). Double-headed arrows indicate the time intervals of teleconnection with the local climate during summer, the entire year, and 3-month running mean values. **b**, High collinearity between DJF NINO3.4 and other ENSO indices, illustrating that the DJF NINO3.4 index can represent ENSO. **c**, Teleconnections measured as partial correlation coefficients between detrended DJF NINO3.4 and population-weighted local temperature and precipitation during different time intervals in 57 tropical countries. The correlation coefficient ranges from negative values (blue) to positive values (red). Significant correlations (BH-adjusted $P \leq 0.05$) are denoted by an asterisk. TCA: Turks and Caicos Islands; VIR: United States Virgin Islands; VCT: Saint Vincent and the Grenadines.

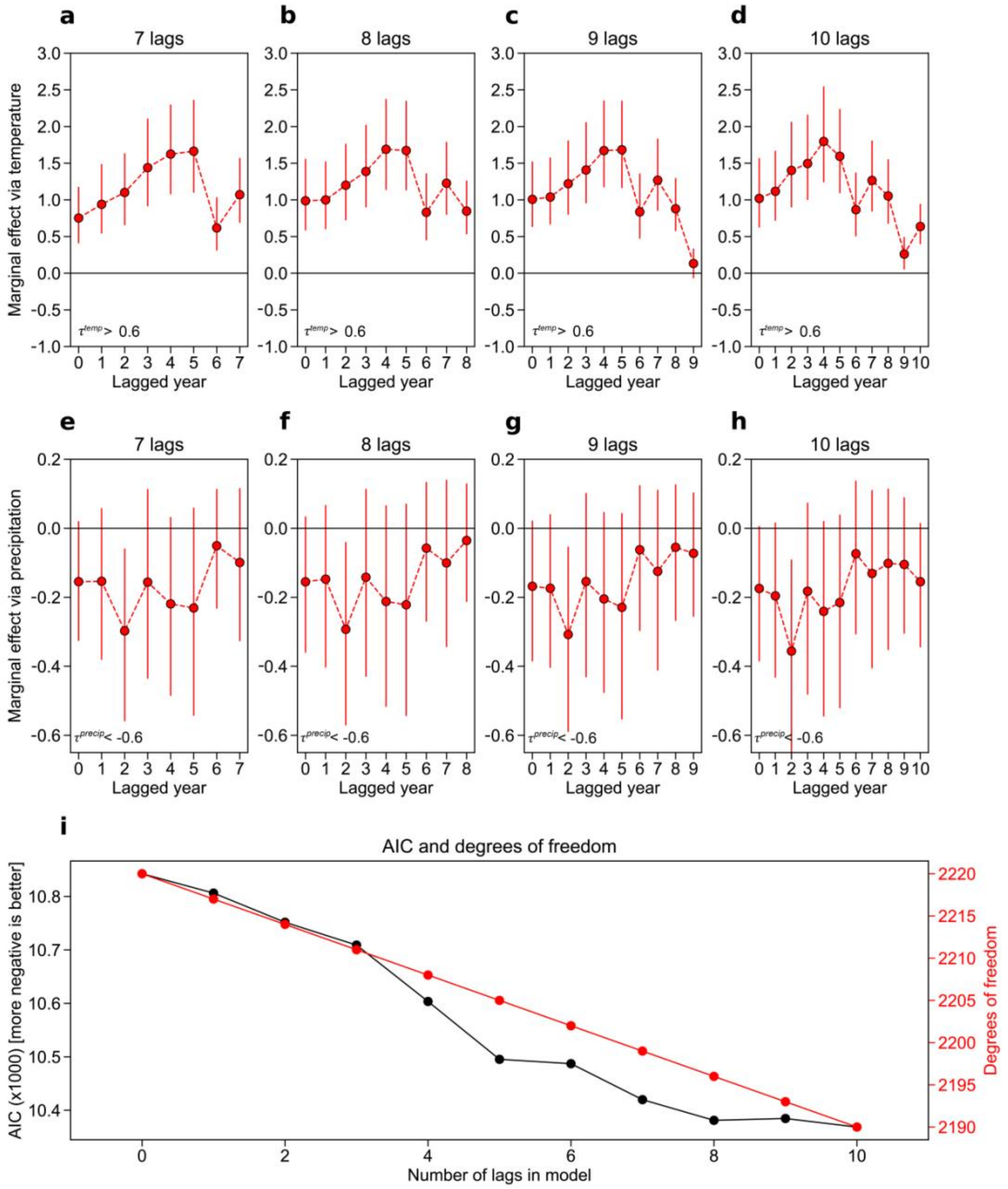

Supplementary Fig. 3 Robustness of the main regression results to additional lags. **a**–**h**, Regression results for countries with ENSO–temperature teleconnections greater than or equal to 0.6 (**a**–**d**) and countries with ENSO–precipitation teleconnections less than or equal to -0.6 (**e**–**h**), estimated with 7, 8, 9, and 10 lags in the regression model. Confidence intervals were generated by bootstrap resampling as in the main analysis. The effect of ENSO on dengue cases in a given lagged year generally exhibits a turning point in the second year, indicating that the lagged impact of ENSO on dengue persists for 2 years. Although the effect of ENSO via temperature peaks in the fifth year, it might be confoundingly influenced by the ENSO cycles of 4 to 6 years and the immune system. **i**, Akaike Information Criterion (AIC) values for regression models with lags from 1 to 10 (black line) and the corresponding number of degrees of freedom (red line). Smaller AIC represents a better-fitted model, but 2 lags are more stable and rational than 5 lags.

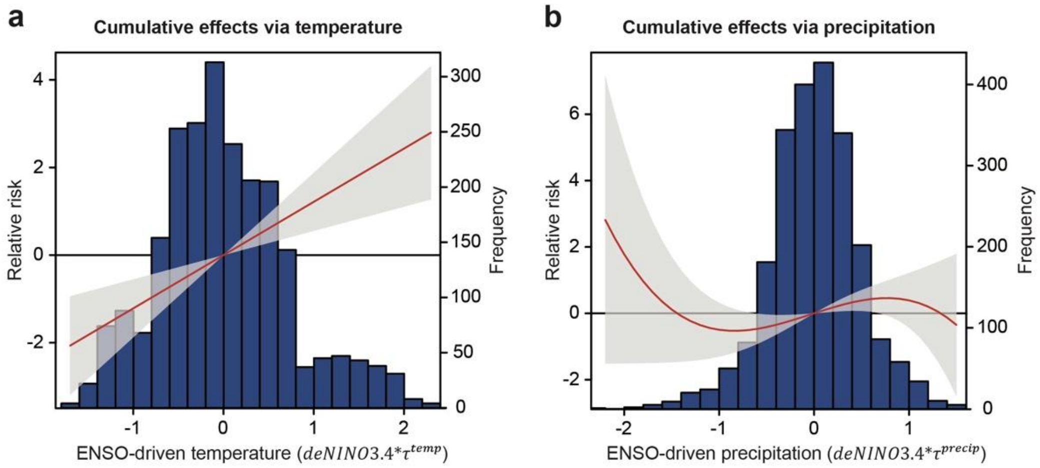

Supplementary Fig. 4 Results of distributed lagged non-linear models (DLNM). The distribution of ENSO-driven temperature and precipitation indicates linear relations between ENSO-driven temperature (**a**) / precipitation (**b**) and dengue risk with only a few extreme values deviating from this pattern, which supports our linear regression model (DLM).

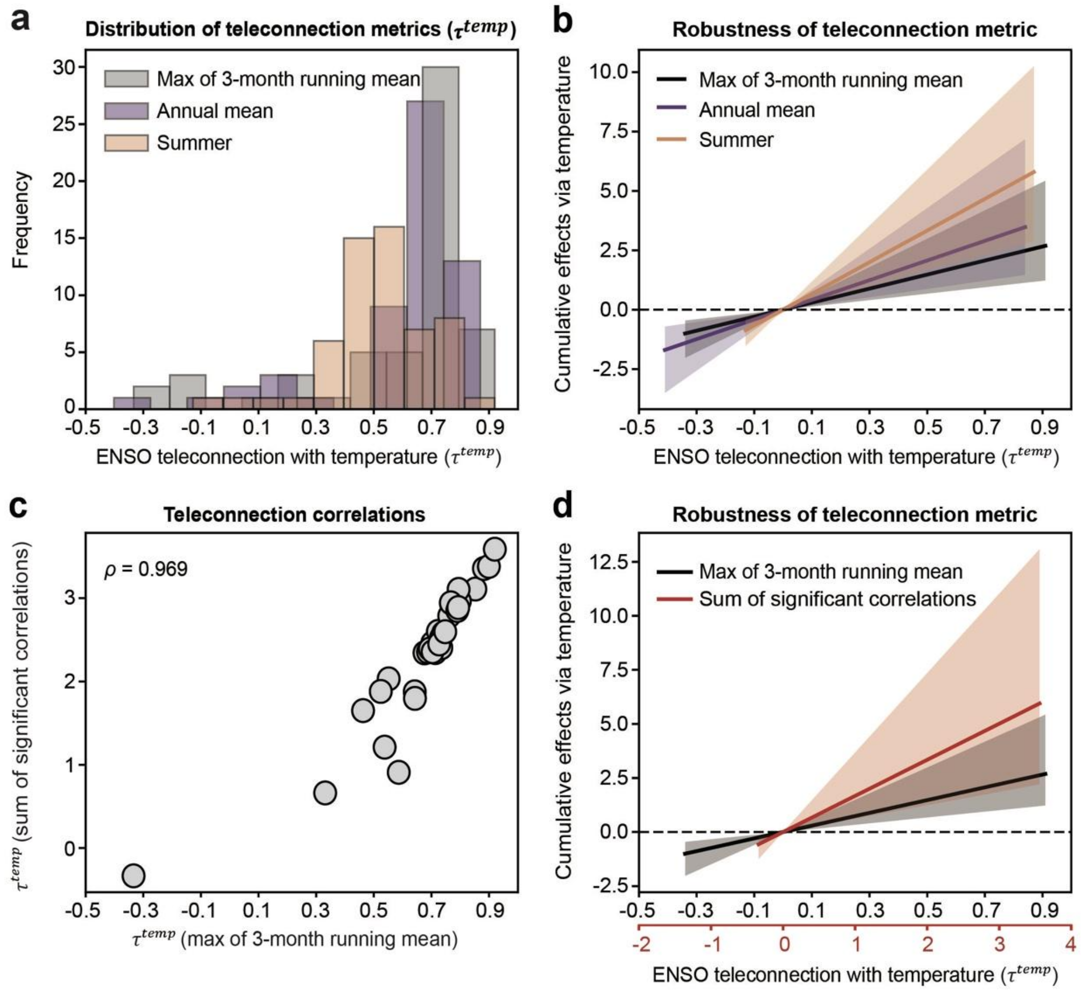

Supplementary Fig. 5 Robustness of ENSO's effects via temperature to alternative teleconnection metrics. a, Distributions of three ENSO teleconnection metrics for 57 countries, including the maximum teleconnection with 3-month running mean temperature (grey), teleconnection with annual mean temperature (purple), and teleconnection with summertime mean temperature (orange). **b**, Cumulative effect of ENSO on dengue cases via temperature using three teleconnection metrics. **c**, Relationship between teleconnections from our main analysis (max of 3-month running mean) and the sum of all statistically significant teleconnections (annual mean + summer). Each point represents teleconnections for a country. Rho denotes the Spearman's rank correlation coefficient between the two teleconnections. **d**, Cumulative effect of ENSO on dengue cases via temperature using the original teleconnection metric (black) and the summed teleconnection metric (red). In **b** and **d**, solid lines denote means and shading indicates 95% confidence intervals across 1,000 bootstrap iterations, as in the main analysis. Results show that ENSO's effects on dengue via temperature are robust across different teleconnection metrics.

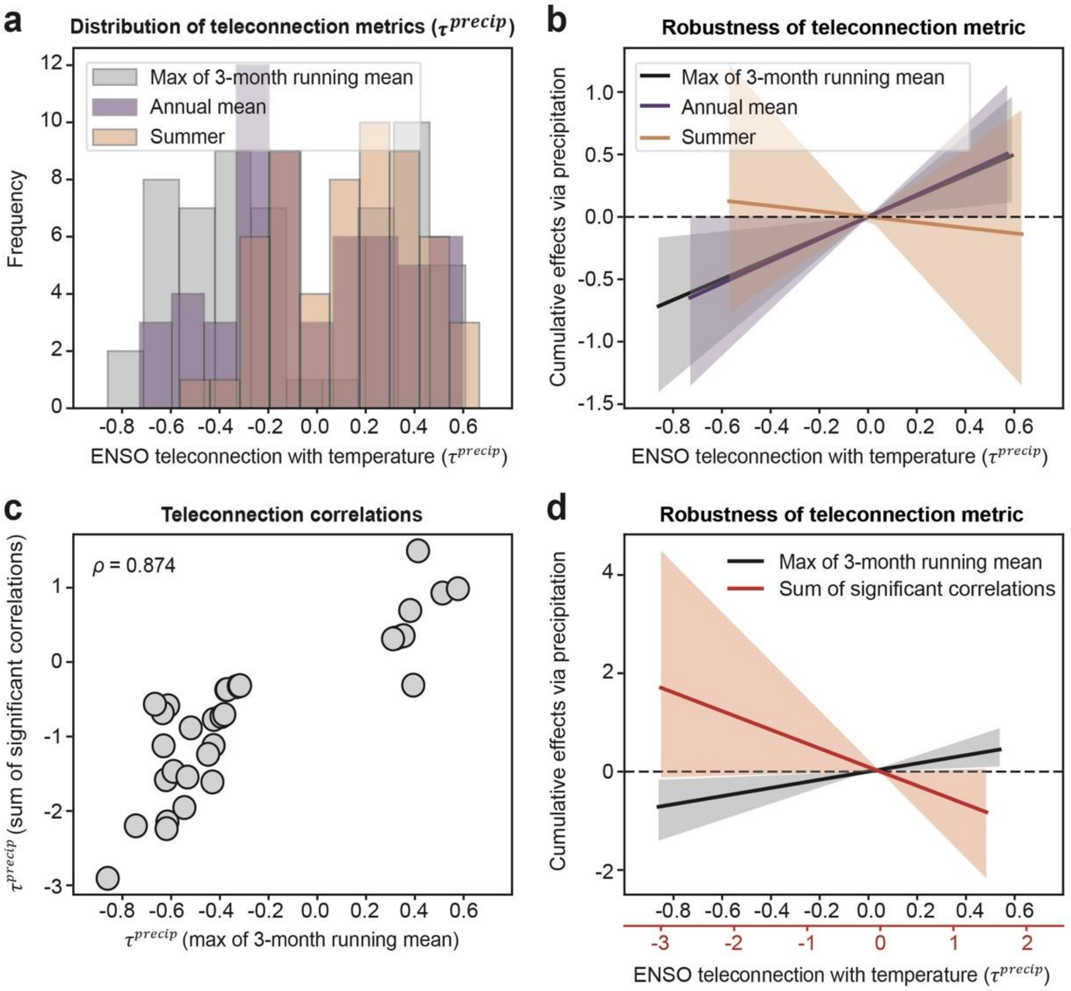

Supplementary Fig. 6 Robustness of ENSO's effects via precipitation to alternative teleconnection metrics. a, Distributions of three ENSO teleconnection metrics for 57 countries, including the maximum teleconnection with 3-month running mean precipitation (grey), teleconnection with annual mean precipitation (purple), and teleconnection with summertime mean precipitation (orange). **b**, Cumulative effect of ENSO on dengue cases via precipitation using three teleconnection metrics. **c**, Relationship between teleconnections from our main analysis (max of 3-month running mean) and the sum of all statistically significant teleconnections (annual mean + summer). Each point represents teleconnections for a country. Rho denotes the Spearman's rank correlation coefficient between the two teleconnections. **d**, Cumulative effect of ENSO on dengue cases via precipitation using the original teleconnection metric (black) and the summed teleconnection metric (red). In **b** and **d**, solid lines denote means and shading indicates 95% confidence intervals across 1,000 bootstrap iterations, as in the main analysis. Results suggest that ENSO's effects on dengue via precipitation are insignificant when using the teleconnection with summertime precipitation.

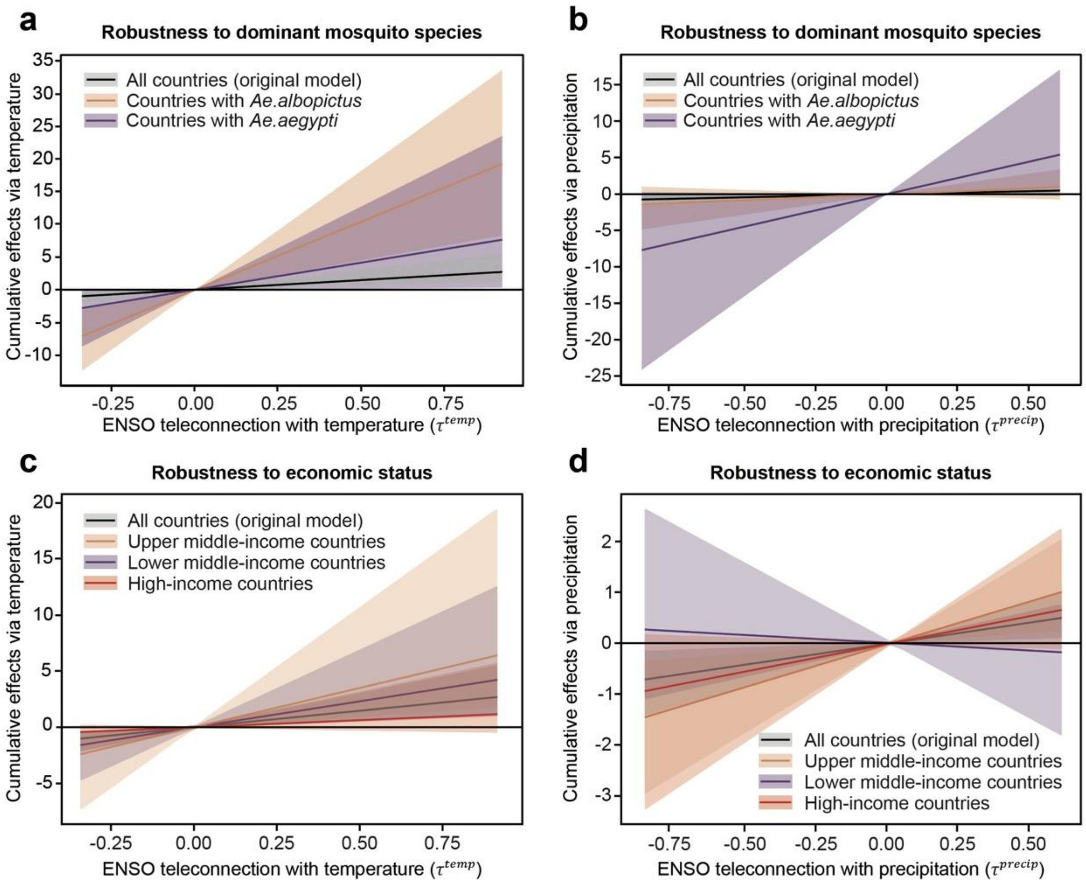

Supplementary Fig. 7 Robustness of main regression results to mosquito species and economic status. The black line denotes the cumulative effects of ENSO on dengue via temperature (**a**,**c**) and precipitation (**b**,**d**) for all countries (original model). **a**–**b**, Regression results of countries grouped by the dominant mosquito species. Countries where the dominant vector is *Ae.albopictus* include Belize, Colombia, Ecuador, El Salvador, Guatemala, Jamaica, Malaysia, Nicaragua, Panama, Peru, and Venezuela. Countries where the dominant vector is *Ae.aegypti* include Bolivia, Brazil, Cambodia, Costa Rica, Dominican Republic, Honduras, Laos, Mexico, Philippines, Singapore, Thailand, and Vietnam. The dominant mosquito species in each country is detected by the best simulated mathematic models for climate responses of mosquito species[2]. Shading indicates the 95% confidence intervals from bootstrap resampling by country, as in the main analysis. **c**–**d**, Regression results of countries grouped by the income level. Countries are classified based on income level in 2020 according to the World Bank definition. ENSO's effects on dengue via temperature are robust across income groups (**c**), while ENSO's effects on dengue via precipitation are insignificant in the lower middle-income group (**d**).

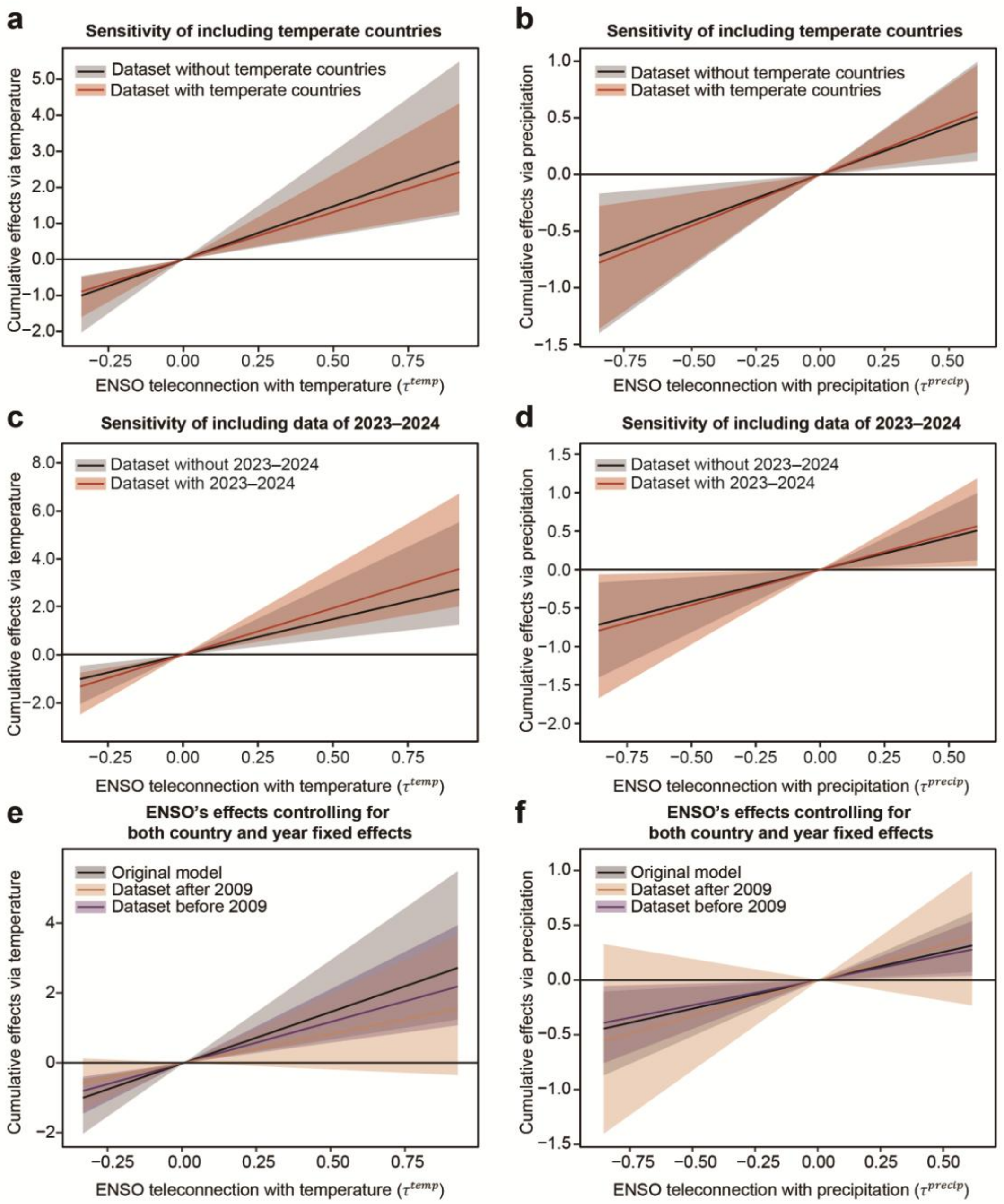

Supplementary Fig. 8 Sensitivity of main regression results to including temperate countries (a–b) and the recent years (c–d) and controlling for both country and year fixed effects (e–f). a–d, ENSO's effects when including temperate countries (**a–b**) and the recent years 2023 and 2024 (**c–d**). The black line denotes the cumulative effects of ENSO on dengue based on the original model, and the red line denotes results when including temperate countries (China, USA, Chile, Argentina) or the recent years 2023 and 2024. Shading indicates the 95% confidence intervals from bootstrap resampling, as in the main analysis. **e–f**, ENSO's effects when controlling for both country and year fixed effects. As the WHO case definition was updated in 2009, we split the dataset by 2009 to control for both country fixed effects and effects of major year-varying confounders. Black lines denote results from the original model, orange lines denote regression results based on the dataset after 2009, and purple lines

denote regression results based on the dataset before 2009. Results show that the change in case definition has reduced the magnitude of ENSO’s effects via temperature by ~43% and increased that via precipitation by ~25%, indicating that changes in case definition might affect the magnitude of ENSO’s effects on dengue epidemics.

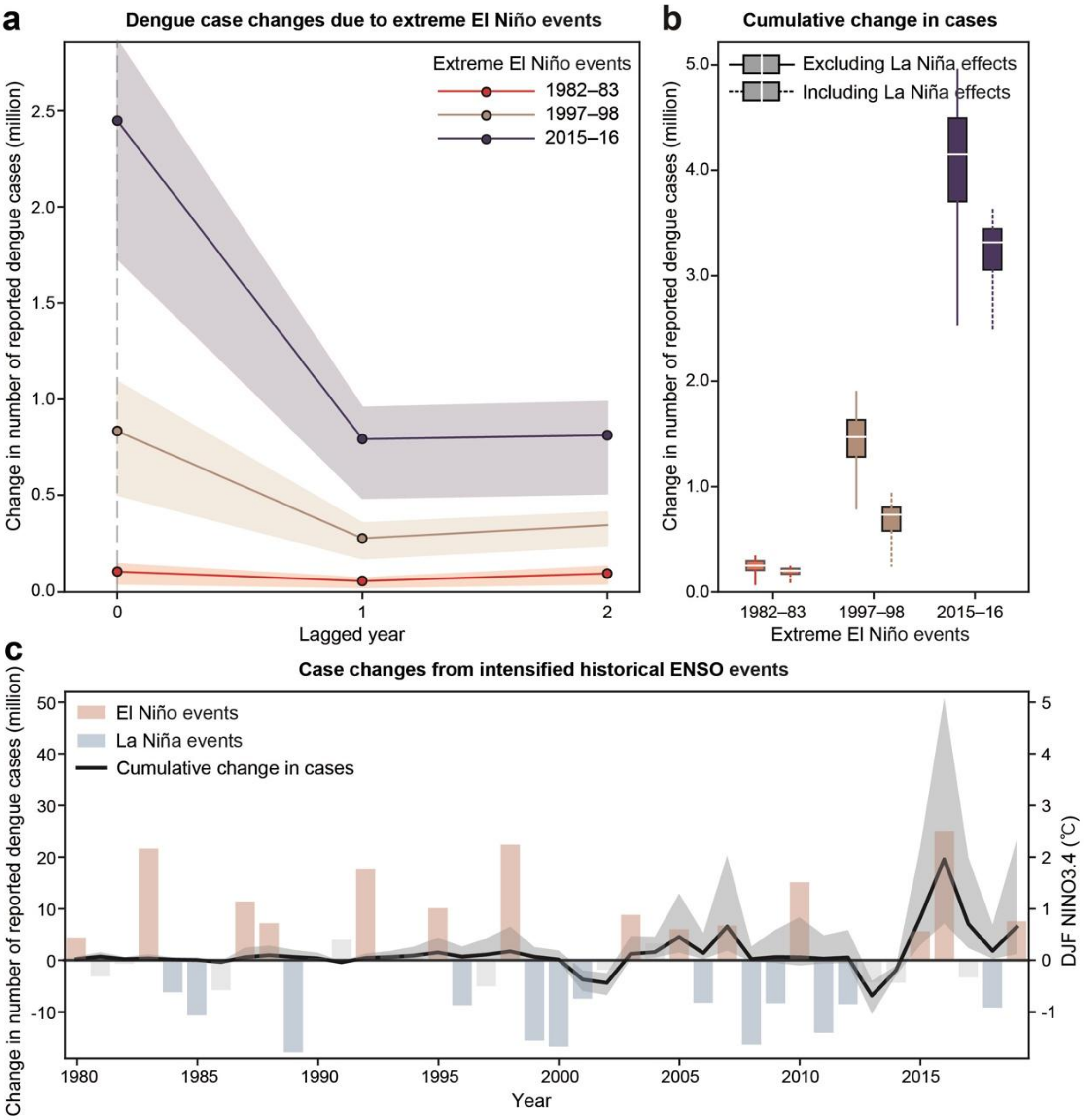

Supplementary Fig. 9 Dengue increases from historical ENSO events. a, Dengue increases from the four strongest El Niño events in 1982–83, 1997–98, 2015–16. Changes in dengue cases are calculated as the changes in global dengue cases due to ENSO-temperature and ENSO-precipitation teleconnections, compared to a baseline scenario without the El Niño event (DJF NINO3.4 = 0) (Methods). The cumulative effects of each El Niño event are represented as the sum of changes in the occurrence year *t* (lag 0), the following year *t*+1 (lag 1), and the second year *t*+2 (lag 2) after the event. **b**, Boxplots display the cumulative change in global dengue cases, with solid lines representing data excluding the influence of the first subsequent La Niña event and dashed lines including it. **c,** Change in dengue cases due to one-unit intensified ENSO events during 1980–2019. Cumulative change in dengue cases is shaped by the changes due to one-unit intensified ENSO events in the current year (*t*), the previous year (*t*-1), and two years prior (*t*-2). Shade indicates 95% CIs from bootstrapping of regression coefficients. Over the whole period, we estimated an average increase of 183.2% in dengue cases across 57 countries due to one-unit intensified ENSO events.

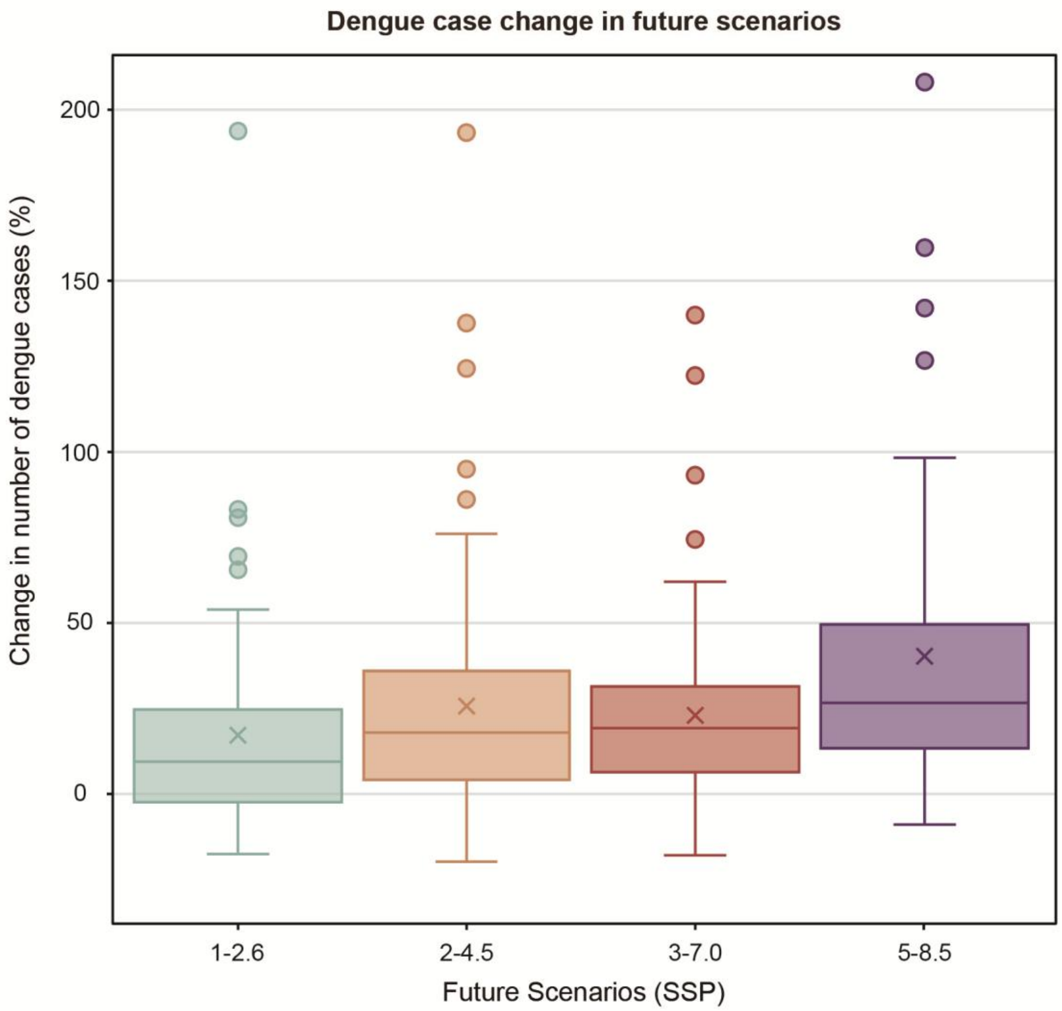

Supplementary Fig. 10 Change in the number of dengue cases in future scenarios. Boxplots show higher increases in the number of dengue cases under SSP5-8.5 compared to other scenarios.

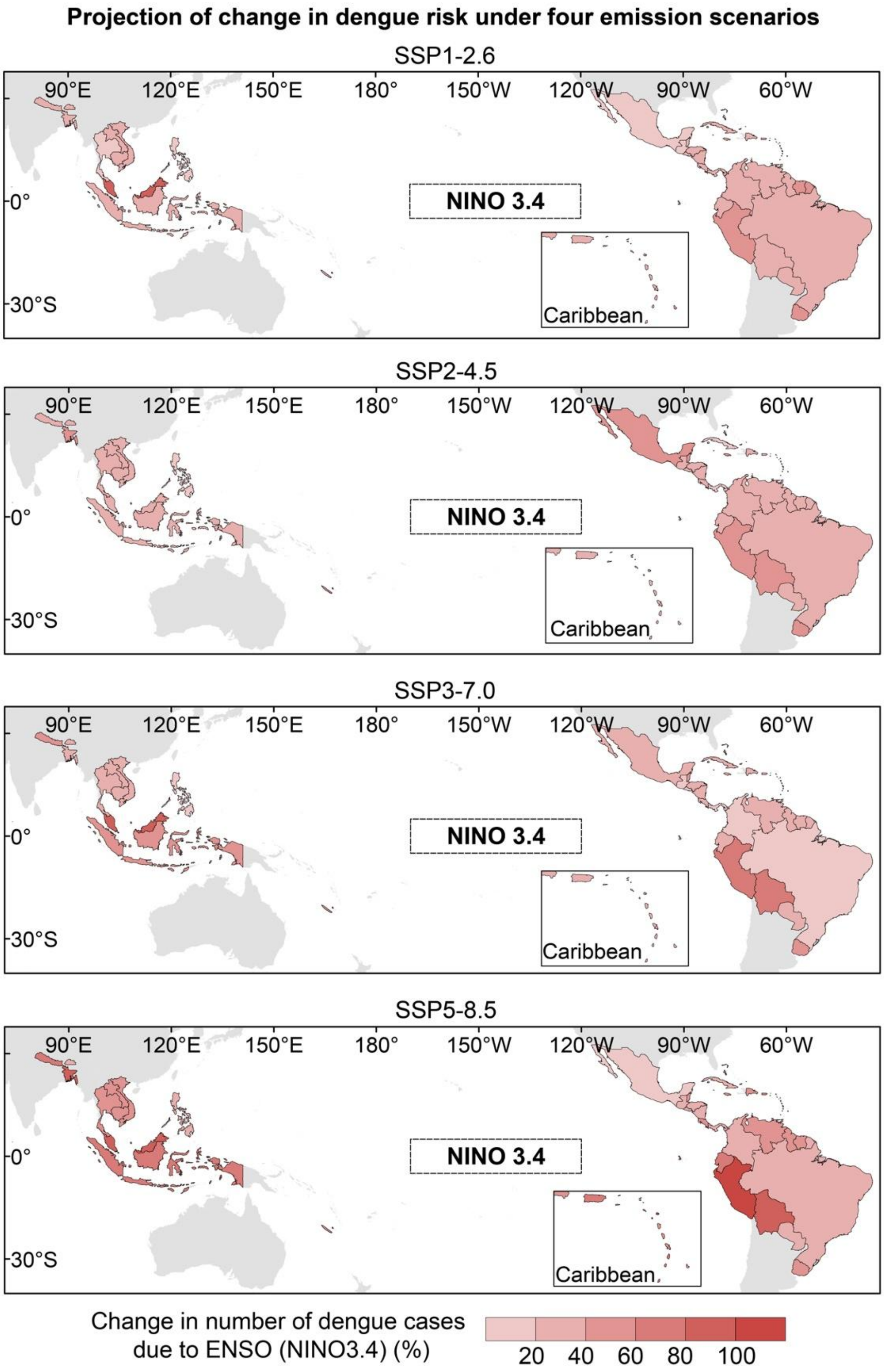

Supplementary Fig. 11 Change in country-level dengue risk due to ENSO evolution under four emission scenarios in 2020–2099. Changes in the number of dengue cases are estimated based on regression coefficients and the median change rates of ENSO amplitude and country-level teleconnections across CMIP6 simulations.

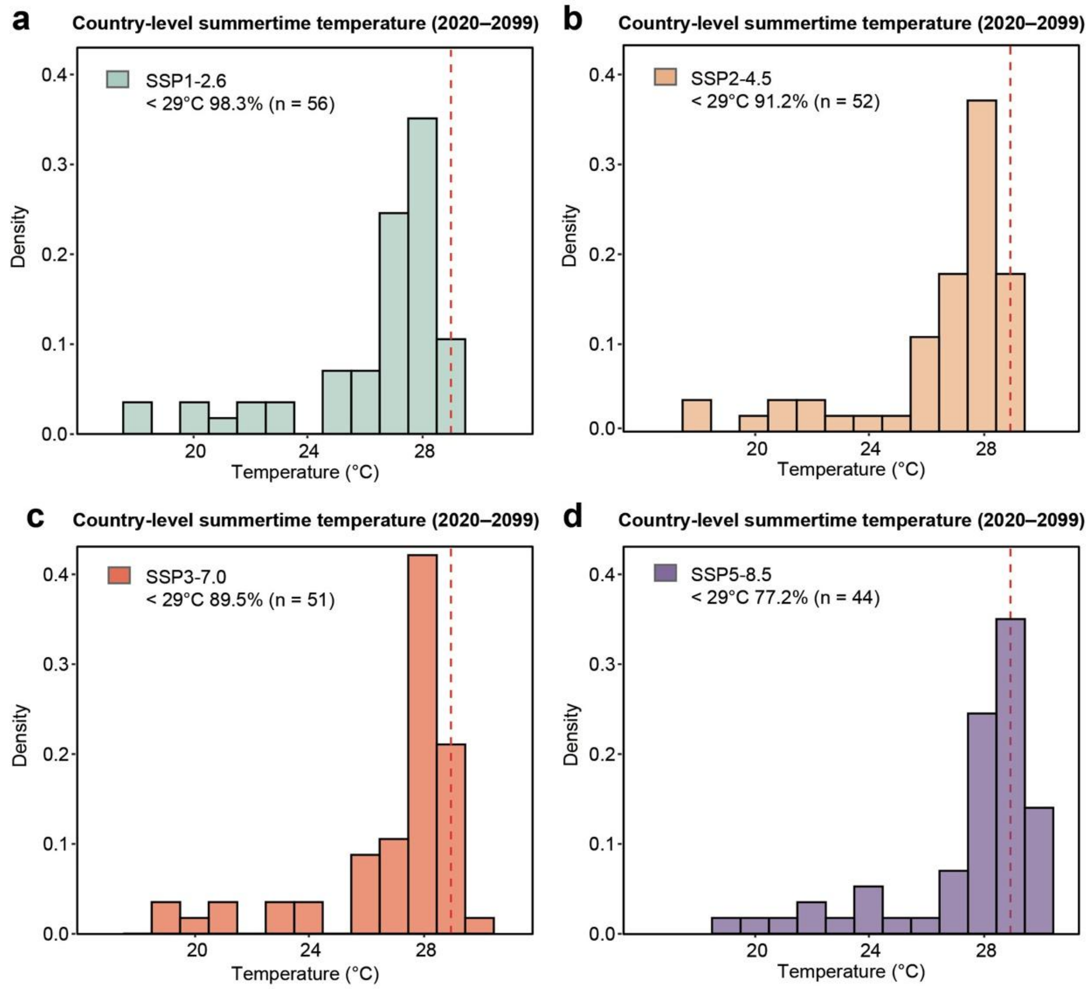

Supplementary Fig. 12 Distribution of country-level summertime temperatures in 2020–2099. The histogram represents the frequency distribution of summertime average temperatures in the future (2020–2099) for each country under the SSP1-2.6 (**a**), SSP2-4.5 (**b**), SSP3-7.0 (**c**), and SSP5-8.5 (**d**) scenarios. The summertime average temperature was calculated as the average value of monthly temperature during summer between 2020 and 2099. Monthly temperatures were debiased from the CMIP predictions based on observed temperatures during 1980–2019 (Methods). The red dashed line denotes the thermal optima for dengue mosquitoes (29°C). The percentage of countries with summertime temperatures below the thermal optima and the number of countries (in parentheses) are given for each scenario, indicating the validity of our predicted dengue risk. We also assessed the average value of annual maximum temperatures between 2020 and 2099 and observed 84.2%, 77.2%, 64.9%, and 35.1% of countries which are likely below the thermal optima under SSP1-2.6, SSP2-4.5, SSP3-7.0, and SSP5-8.5 scenarios, respectively.

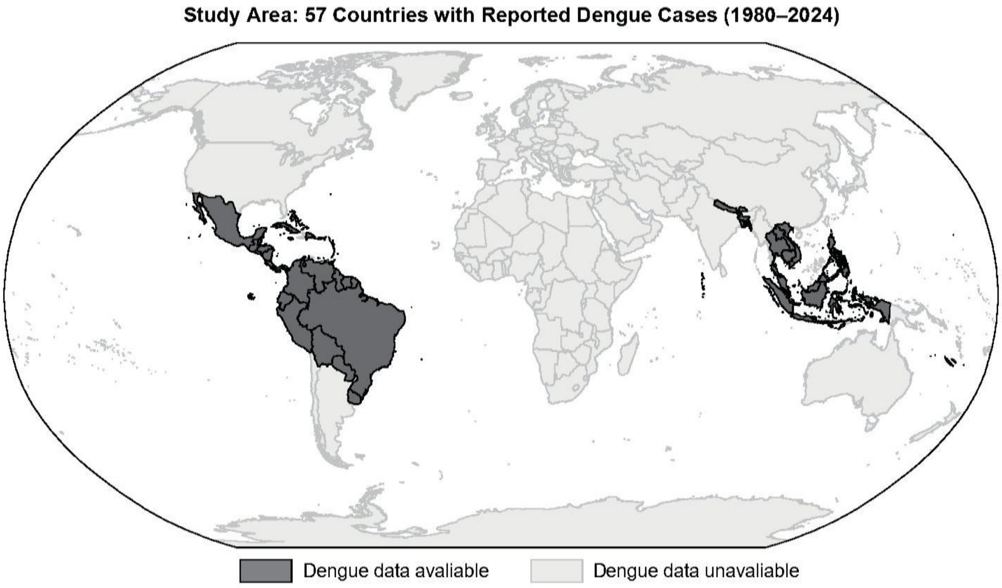

Supplementary Fig. 13 Map of dengue case data availability from 1980 to 2024.

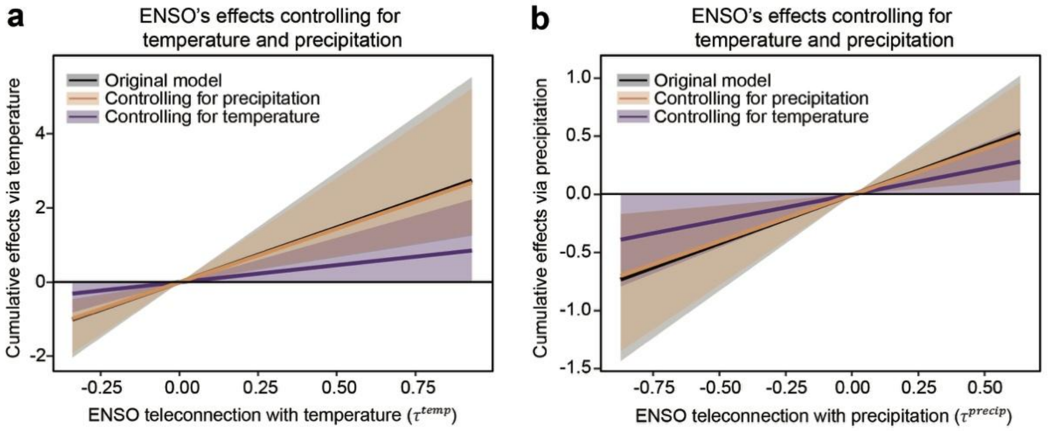

Supplementary Fig. 14 ENSO's effects via temperature (a) and precipitation (b) when controlling for temperature and precipitation. Black lines denote results from the original model, orange lines denote results with the addition of quadratic terms for country-level annual mean temperature in the model, and purple lines denote results with the addition of quadratic terms for the country-level annual average of monthly cumulative precipitation in the model. We did not include linear terms for temperature and precipitation due to the high collinearity between linear and quadratic terms. Shading indicates the 95% confidence intervals from bootstrap resampling, as in the main analysis. Controlling for local temperature or precipitation in our regression did not alter the effects of ENSO, suggesting that our results are distinct from those simulations and projections based on temperature and precipitation.

Supplementary Tables

Supplementary Table 1 Summary of the original regression model and alternative models.

	Dependent variable: log (case)					
	Original model	Annual teleconnections	Summer teleconnections	Winter teleconnections	Including temperate countries	Involve categorical variables
	(1)	(2)	(3)	(4)	(5)	(6)
$\boldsymbol{NINO3.4_t(\beta_0)}$	-0.63*** (0.15)	-0.87*** (0.22)	-1.15*** (0.25)	-0.42*** (0.12)	-0.54*** (0.13)	-1.24*** (0.19)
$\boldsymbol{NINO3.4_{t-1}(\beta_1)}$	-0.72*** (0.13)	-0.89*** (0.17)	-1.16*** (0.20)	-0.57*** (0.11)	-0.68*** (0.11)	-0.68*** (0.13)
$\boldsymbol{NINO3.4_{t-2}(\beta_2)}$	-0.82*** (0.14)	-1.04*** (0.18)	-1.35*** (0.22)	-0.63*** (0.11)	-0.75*** (0.12)	-0.77*** (0.13)
$\boldsymbol{deNINO3.4_t * \tau_i^{temp}(\theta_0^{temp})}$	0.91*** (0.22)	1.31*** (0.33)	2.06*** (0.47)	0.81*** (0.25)	0.81*** (0.20)	0.84*** (0.22)
$\boldsymbol{deNINO3.4_{t-1} * \tau_i^{temp}(\theta_1^{temp})}$	0.80*** (0.20)	1.06*** (0.26)	1.80*** (0.38)	0.79*** (0.23)	0.75*** (0.17)	0.72*** (0.19)
$\boldsymbol{deNINO3.4_{t-2} * \tau_i^{temp}(\theta_2^{temp})}$	0.93*** (0.20)	1.26*** (0.28)	2.01*** (0.40)	0.88*** (0.24)	0.84*** (0.18)	0.85*** (0.20)
$\boldsymbol{deNINO3.4_t * \tau_i^{precip}(\theta_0^{precip})}$	0.23* (0.12)	0.24. (0.15)	-0.05 (0.22)	0.16 (0.16)	0.26* (0.11)	0.22. (0.12)
$\boldsymbol{deNINO3.4_{t-1} * \tau_i^{precip}(\theta_1^{precip})}$	0.19. (0.11)	0.18 (0.14)	-0.16 (0.19)	0.15 (0.15)	0.23* (0.10)	0.18 (0.11)
$\boldsymbol{deNINO3.4_{t-2} * \tau_i^{precip}(\theta_2^{precip})}$	0.37** (0.12)	0.45** (0.15)	0.12 (0.21)	0.30* (0.15)	0.39*** (0.11)	0.35** (0.12)
Country-fixed effect	Yes	Yes	Yes	Yes	Yes	Yes
El Niño event	-	-	-	-	-	1.53*** (0.19)
La Niña event	-	-	-	-	-	-0.35* (0.14)
Teleconnection metrics	Running mean	year	summer	winter	Running mean	Running mean
Observations	57*40	57*40	57*40	57*40	61*40	57*40
Adjusted R^2	0.63	0.63	0.64	0.62	0.62	0.64

The numbers outside the parentheses are regression coefficients, and the numbers within parentheses following each coefficient represent the standard errors adjusted by the Newey-West method. The significant level is assessed by a two-tailed test. $***P \leq 0.001$; $**P \leq 0.01$; $*P \leq 0.05$; $.P \leq 0.1$

Supplementary Table 2 Fitness of linear and non-linear models.

Model	Adjusted R^2	AIC	BIC
Linear distributed lag model (DLM)	0.63	10751.71	11135.75
Non-linear model	0.63	10740.66	11193.49

The non-linear model was constructed by adding spline functions for ENSO-driven temperature (df = 2) and precipitation (df = 4) to our linear distributed lag model (DLM). The similar fitness metrics of linear and non-linear models indicate that most observations adhere to a linear relationship.

Supplementary Table 3 Correlation between the change in the dominant circulation strain and reported dengue cases from 1990 to 2019.

Country	Log (case) ~ Strain change (0/1)	*P* value
Brazil	0.27	0.12
Cambodia	-0.13	0.57
Thailand	-0.09	0.58
Singapore	0.28	0.24
Malaysia	0.51**	0.00
Vietnam	0.16	0.46
Philippines	0.09	0.67
Indonesia	0.34	0.13
Mexico	0.68**	0.00

We analysed the Pearson correlation coefficients based on available serotype data and found insignificant correlations ($P > 0.05$) in 7 out of 9 countries, suggesting that changes in the dominant circulation strain may not influence our results. The serotype with the highest proportion was identified as the circulating serotype in year *t* and year *t*+1. A binary sequence (0/1) was utilized to demote changes in the dominant circulation strain in year *t*+1. Significance was assessed using two-tailed tests.

**$P \leq 0.01$

Supplementary Table 4 CMIP6 models and their ensemble members used for SSP1-2.6, SSP2-4.5, SSP3-7.0, and SSP5-8.5 emission scenarios in this study.

**Model**	**Ensemble**	**SSP available (Bold represents high-skill)**
BCC-CSM2-MR	r1i1p1f1	**SSP1-2.6, SSP2-4.5, SSP3-7.0, SSP5-8.5**
CAMS-CSM1-0	r1i1p1f1	**SSP1-2.6, SSP2-4.5, SSP3-7.0, SSP5-8.5**
CanESM5	r1i1p1f1	**SSP1-2.6, SSP2-4.5, SSP3-7.0, SSP5-8.5**
CanESM5-1	r1i1p1f1	**SSP1-2.6, SSP2-4.5, SSP3-7.0, SSP5-8.5**
CanESM5-CanOE	r1i1p2f1	**SSP1-2.6, SSP2-4.5, SSP3-7.0, SSP5-8.5**
CAS-ESM2-0	r1i1p1f1	**SSP2-4.5, SSP3-7.0, SSP5-8.5**
CESM2	r4i1p1f1	SSP1-2.6, SSP2-4.5, SSP3-7.0, SSP5-8.5
	r1i1p1f1	**SSP1-2.6,** SSP2-4.5, **SSP3-7.0, SSP5-8.5**
CESM2-WACCM	r2i1p1f1	SSP2-4.5, SSP5-8.5
	r3i1p1f1	SSP2-4.5, SSP5-8.5
CIESM	r1i1p1f1	**SSP1-2.6, SSP2-4.5, SSP5-8.5**
CMCC-CM2-SR5	r1i1p1f1	**SSP1-2.6,** SSP2-4.5, **SSP3-7.0, SSP5-8.5**
CMCC-ESM2	r1i1p1f1	SSP1-2.6, SSP2-4.5, SSP3-7.0, SSP5-8.5
CNRM-CM6-1	r1i1p1f2	**SSP1-2.6, SSP2-4.5, SSP3-7.0, SSP5-8.5**
CNRM-CM6-1-HR	r1i1p1f2	**SSP1-2.6, SSP2-4.5, SSP3-7.0, SSP5-8.5**
CNRM-ESM2-1	r1i1p1f2	**SSP1-2.6, SSP2-4.5, SSP3-7.0, SSP5-8.5**
E3SM-1-1	r1i1p1f1	**SSP5-8.5**
EC-Earth3	r1i1p1f1	**SSP1-2.6, SSP2-4.5, SSP3-7.0, SSP5-8.5**
EC-Earth3-CC	r1i1p1f1	**SSP2-4.5, SSP5-8.5**
EC-Earth3-Veg	r1i1p1f1	**SSP1-2.6, SSP2-4.5, SSP3-7.0, SSP5-8.5**
EC-Earth3-Veg-LR	r1i1p1f1	**SSP1-2.6, SSP2-4.5, SSP3-7.0, SSP5-8.5**
FGOALS-f3-L	r1i1p1f1	SSP1-2.6, SSP2-4.5, SSP3-7.0, SSP5-8.5
FGOALS-g3	r1i1p1f1	**SSP1-2.6, SSP2-4.5, SSP3-7.0, SSP5-8.5**
FIO-ESM-2-0	r1i1p1f1	**SSP1-2.6, SSP2-4.5, SSP5-8.5**
GFDL-CM4	r1i1p1f1	**SSP2-4.5, SSP5-8.5**
GFDL-ESM4	r1i1p1f1	**SSP1-2.6, SSP2-4.5, SSP3-7.0, SSP5-8.5**
GISS-E2-1-G	r1i1p1f2	**SSP1-2.6, SSP2-4.5, SSP3-7.0, SSP5-8.5**
HadGEM3-GC31-LL	r1i1p1f3	**SSP1-2.6, SSP2-4.5, SSP5-8.5**
HadGEM3-GC31-MM	r1i1p1f3	**SSP1-2.6, SSP5-8.5**
INM-CM4-8	r1i1p1f1	SSP1-2.6, **SSP2-4.5, SSP3-7.0, SSP5-8.5**
INM-CM5-0	r1i1p1f1	**SSP1-2.6, SSP2-4.5, SSP3-7.0, SSP5-8.5**
IPSL-CM6A-LR	r1i1p1f1	**SSP1-2.6**, **SSP2-4.5,** SSP3-7.0, SSP5-8.5
IPSL-CM5A2-INCA	r1i1p1f1	**SSP1-2.6, SSP3-7.0**
KACE-1-0-G	r1i1p1f1	**SSP1-2.6, SSP2-4.5, SSP3-7.0, SSP5-8.5**
KIOST-ESM	r1i1p1f1	**SSP1-2.6,** SSP2-4.5, **SSP5-8.5**
MCM-UA-1-0	r1i1p1f2	**SSP1-2.6, SSP2-4.5, SSP3-7.0, SSP5-8.5**

MIROC6	r1i1p1f1	**SSP1-2.6, SSP2-4.5,** SSP3-7.0, SSP5-8.5
	r2i1p1f1	SSP1-2.6, SSP2-4.5, SSP3-7.0, SSP5-8.5
	r3i1p1f1	**SSP1-2.6, SSP2-4.5, SSP3-7.0,** SSP5-8.5
	r4i1p1f1	SSP1-2.6, SSP2-4.5, SSP3-7.0, SSP5-8.5
	r5i1p1f1	**SSP1-2.6, SSP2-4.5, SSP3-7.0, SSP5-8.5**
	r6i1p1f1	**SSP1-2.6, SSP2-4.5, SSP3-7.0, SSP5-8.5**
	r7i1p1f1	**SSP1-2.6, SSP2-4.5, SSP3-7.0, SSP5-8.5**
	r8i1p1f1	**SSP1-2.6,** SSP2-4.5, SSP3-7.0, SSP5-8.5
	r9i1p1f1	**SSP1-2.6, SSP2-4.5, SSP3-7.0,** SSP5-8.5
	r10i1p1f1	**SSP1-2.6, SSP2-4.5, SSP3-7.0,** SSP5-8.5
	r11i1p1f1	**SSP1-2.6, SSP2-4.5, SSP3-7.0, SSP5-8.5**
	r12i1p1f1	**SSP1-2.6, SSP2-4.5, SSP3-7.0,** SSP5-8.5
	r13i1p1f1	**SSP1-2.6, SSP2-4.5, SSP3-7.0, SSP5-8.5**
	r14i1p1f1	SSP1-2.6, SSP2-4.5, SSP3-7.0, SSP5-8.5
	r15i1p1f1	**SSP1-2.6, SSP2-4.5, SSP3-7.0, SSP5-8.5**
	r16i1p1f1	**SSP1-2.6,** SSP2-4.5, SSP3-7.0, SSP5-8.5
	r17i1p1f1	**SSP1-2.6, SSP2-4.5,** SSP3-7.0, **SSP5-8.5**
	r18i1p1f1	**SSP1-2.6, SSP2-4.5,** SSP3-7.0, **SSP5-8.5**
	r19i1p1f1	SSP1-2.6, SSP2-4.5, **SSP3-7.0,** SSP5-8.5
	r20i1p1f1	**SSP1-2.6,** SSP2-4.5, **SSP3-7.0,** SSP5-8.5
	r21i1p1f1	**SSP1-2.6, SSP2-4.5, SSP3-7.0,** SSP5-8.5
	r22i1p1f1	**SSP1-2.6, SSP2-4.5, SSP3-7.0, SSP5-8.5**
	r23i1p1f1	**SSP1-2.6, SSP2-4.5, SSP3-7.0, SSP5-8.5**
	r24i1p1f1	SSP1-2.6, **SSP2-4.5,** SSP3-7.0, SSP5-8.5

	r25i1p1f1	**SSP1-2.6, SSP2-4.5, SSP3-7.0, SSP5-8.5**
	r26i1p1f1	**SSP1-2.6, SSP2-4.5, SSP3-7.0,** SSP5-8.5
	r27i1p1f1	SSP1-2.6, **SSP2-4.5,** SSP3-7.0, SSP5-8.5
	r28i1p1f1	**SSP1-2.6, SSP2-4.5, SSP3-7.0, SSP5-8.5**
	r29i1p1f1	**SSP1-2.6, SSP2-4.5, SSP3-7.0, SSP5-8.5**
	r30i1p1f1	**SSP1-2.6, SSP2-4.5, SSP3-7.0, SSP5-8.5**
	r31i1p1f1	**SSP1-2.6, SSP2-4.5, SSP3-7.0, SSP5-8.5**
	r32i1p1f1	**SSP1-2.6,** SSP2-4.5, SSP3-7.0, SSP5-8.5
	r33i1p1f1	SSP1-2.6, SSP2-4.5, SSP3-7.0, SSP5-8.5
	r34i1p1f1	**SSP1-2.6, SSP2-4.5,** SSP3-7.0, SSP5-8.5
	r35i1p1f1	**SSP1-2.6,** SSP2-4.5, **SSP3-7.0, SSP5-8.5**
	r36i1p1f1	**SSP1-2.6, SSP2-4.5, SSP3-7.0, SSP5-8.5**
	r37i1p1f1	**SSP1-2.6, SSP2-4.5, SSP3-7.0,** SSP5-8.5
	r38i1p1f1	SSP1-2.6, **SSP2-4.5, SSP3-7.0, SSP5-8.5**
	r39i1p1f1	**SSP1-2.6, SSP2-4.5, SSP3-7.0, SSP5-8.5**
	r40i1p1f1	**SSP1-2.6, SSP2-4.5, SSP3-7.0, SSP5-8.5**
	r41i1p1f1	**SSP1-2.6, SSP2-4.5, SSP3-7.0, SSP5-8.5**
	r42i1p1f1	**SSP1-2.6, SSP2-4.5,** SSP3-7.0, SSP5-8.5
	r43i1p1f1	**SSP1-2.6, SSP2-4.5, SSP3-7.0, SSP5-8.5**
	r44i1p1f1	SSP1-2.6, SSP2-4.5, SSP3-7.0, SSP5-8.5
	r45i1p1f1	SSP1-2.6, SSP2-4.5, SSP3-7.0, SSP5-8.5
	r46i1p1f1	**SSP1-2.6, SSP2-4.5,** SSP3-7.0, SSP5-8.5
	r47i1p1f1	SSP1-2.6, SSP2-4.5, SSP3-7.0, SSP5-8.5
	r48i1p1f1	**SSP1-2.6,** SSP2-4.5, **SSP3-7.0,** SSP5-8.5
	r49i1p1f1	SSP1-2.6, SSP2-4.5, SSP3-7.0, SSP5-8.5
	r50i1p1f1	**SSP1-2.6, SSP2-4.5, SSP3-7.0, SSP5-8.5**
MIROC-ES2L	r1i1p1f2	SSP1-2.6, SSP2-4.5, SSP3-7.0, SSP5-8.5
	r2i1p1f2	**SSP2-4.5, SSP3-7.0**
	r3i1p1f2	SSP2-4.5, SSP3-7.0
	r4i1p1f2	SSP2-4.5, SSP3-7.0
	r5i1p1f2	SSP2-4.5, SSP3-7.0
	r6i1p1f2	**SSP2-4.5, SSP3-7.0**
	r7i1p1f2	SSP2-4.5, SSP3-7.0
	r8i1p1f2	SSP2-4.5, SSP3-7.0
	r9i1p1f2	SSP2-4.5, SSP3-7.0
	r10i1p1f2	**SSP2-4.5,** SSP3-7.0
	r11i1p1f2	SSP2-4.5
	r12i1p1f2	SSP2-4.5
	r13i1p1f2	SSP2-4.5
	r14i1p1f2	SSP2-4.5
	r15i1p1f2	SSP2-4.5
	r16i1p1f2	SSP2-4.5

	r17i1p1f2	SSP2-4.5
	r18i1p1f2	**SSP2-4.5**
	r19i1p1f2	SSP2-4.5
	r20i1p1f2	SSP2-4.5
	r21i1p1f2	**SSP2-4.5**
	r22i1p1f2	**SSP2-4.5**
	r23i1p1f2	SSP2-4.5
	r24i1p1f2	**SSP2-4.5**
	r25i1p1f2	SSP2-4.5
	r26i1p1f2	SSP2-4.5
	r27i1p1f2	**SSP2-4.5**
	r28i1p1f2	SSP2-4.5
	r29i1p1f2	**SSP2-4.5**
	r30i1p1f2	**SSP2-4.5**
	r1i1p1f1	**SSP1-2.6, SSP2-4.5, SSP3-7.0, SSP5-8.5**
	r2i1p1f1	**SSP2-4.5, SSP3-7.0, SSP5-8.5**
	r3i1p1f1	**SSP3-7.0**
	r4i1p1f1	**SSP3-7.0**
	r5i1p1f1	**SSP3-7.0**
MPI-ESM1-2-HR	r6i1p1f1	**SSP3-7.0**
	r7i1p1f1	**SSP3-7.0**
	r8i1p1f1	**SSP3-7.0**
	r9i1p1f1	**SSP3-7.0**
	r10i1p1f1	**SSP3-7.0**
	r1i1p1f1	**SSP1-2.6, SSP2-4.5, SSP3-7.0, SSP5-8.5**
	r2i1p1f1	**SSP2-4.5, SSP3-7.0**
	r3i1p1f1	**SSP2-4.5, SSP3-7.0**
	r4i1p1f1	**SSP2-4.5, SSP3-7.0**
	r5i1p1f1	**SSP2-4.5, SSP3-7.0**
	r6i1p1f1	**SSP2-4.5, SSP3-7.0**
	r7i1p1f1	**SSP2-4.5, SSP3-7.0**
	r8i1p1f1	**SSP2-4.5, SSP3-7.0**
	r9i1p1f1	**SSP2-4.5, SSP3-7.0**
MPI-ESM1-2-LR	r10i1p1f1	**SSP2-4.5, SSP3-7.0**
	r11i1p1f1	**SSP2-4.5, SSP3-7.0**
	r12i1p1f1	**SSP2-4.5, SSP3-7.0**
	r13i1p1f1	**SSP2-4.5, SSP3-7.0**
	r14i1p1f1	**SSP2-4.5, SSP3-7.0**
	r15i1p1f1	**SSP2-4.5, SSP3-7.0**
	r16i1p1f1	**SSP2-4.5, SSP3-7.0**
	r17i1p1f1	**SSP2-4.5, SSP3-7.0**
	r18i1p1f1	**SSP2-4.5, SSP3-7.0**

	r19i1p1f1	**SSP2-4.5, SSP3-7.0**
	r20i1p1f1	**SSP2-4.5, SSP3-7.0**
	r21i1p1f1	**SSP2-4.5, SSP3-7.0**
	r22i1p1f1	**SSP2-4.5, SSP3-7.0**
	r23i1p1f1	**SSP2-4.5, SSP3-7.0**
	r24i1p1f1	**SSP2-4.5, SSP3-7.0**
	r25i1p1f1	**SSP2-4.5, SSP3-7.0**
	r26i1p1f1	**SSP2-4.5, SSP3-7.0**
	r27i1p1f1	**SSP2-4.5, SSP3-7.0**
	r28i1p1f1	**SSP2-4.5, SSP3-7.0**
	r29i1p1f1	**SSP2-4.5, SSP3-7.0**
	r30i1p1f1	**SSP2-4.5, SSP3-7.0**
	r1i1p1f1	**SSP1-2.6, SSP2-4.5, SSP3-7.0, SSP5-8.5**
	r2i1p1f1	**SSP3-7.0**
MRI-ESM2-0	r3i1p1f1	**SSP3-7.0**
	r4i1p1f1	**SSP3-7.0**
	r5i1p1f1	**SSP3-7.0**
NESM3	r1i1p1f1	**SSP1-2.6, SSP2-4.5, SSP5-8.5**
NorESM2-LM	r1i1p1f1	SSP1-2.6, SSP2-4.5, SSP3-7.0, SSP5-8.5
NorESM2-MM	r1i1p1f1	SSP1-2.6, SSP2-4.5, SSP3-7.0, SSP5-8.5
UKESM1-0-LL	r1i1p1f2	**SSP1-2.6, SSP2-4.5, SSP3-7.0, SSP5-8.5**

Sea surface temperature (“tos”), atmospheric temperature (“tas”), and precipitation (“pr”) were used from each CMIP model. Bolded models are high-skill models we selected based on the simulation of the NINO3.4 index (Methods).

The ensemble column includes the realization index (“r”), initial condition index (“i”), physics version (“p”), and forcing index (“f”). These indices provide a comprehensive description of the setup used in each simulation (e.g. r1i1p1f1).

Supplementary Table 5 Data sources for yearly reported dengue cases from 1980 to 2024.

Country	Sources
Anguilla	PAHO, OpenDengue
Antigua and Barbuda	PAHO, OpenDengue
Argentina	PAHO, OpenDengue
Aruba	PAHO, OpenDengue
Bahamas	PAHO, OpenDengue
Bangladesh	WHO/WPRO, OpenDengue
Barbados	PAHO, OpenDengue
Belize	PAHO, OpenDengue
Bermuda	PAHO, OpenDengue
Bhutan	WHO/WPRO, RCDC, OpenDengue
Bolivia	PAHO, OpenDengue
Brazil	PAHO, OpenDengue
Cambodia	WHO/WPRO, OpenDengue
Cayman Islands	PAHO, OpenDengue
Chile	PAHO, OpenDengue
China	WHO/WPRO, CDC, OpenDengue, other[1]
Colombia	PAHO, OpenDengue
Costa Rica	PAHO, OpenDengue
Cuba	PAHO, OpenDengue
Dominica	PAHO, OpenDengue
Dominican Republic	PAHO, OpenDengue
Ecuador	PAHO, OpenDengue
El Salvador	PAHO, OpenDengue
French Guiana	PAHO, OpenDengue
Grenada	PAHO, OpenDengue
Guadeloupe	PAHO, OpenDengue
Guatemala	PAHO, OpenDengue
Guyana	PAHO, OpenDengue
Honduras	PAHO, OpenDengue
Indonesia	OpenDengue, National Statistics[2]
Jamaica	PAHO, OpenDengue
Laos	WHO/WPRO, OpenDengue
Malaysia	WHO/WPRO, MOH, OpenDengue
Maldives	WHO/WPRO, OpenDengue
Martinique	PAHO, OpenDengue
Mexico	PAHO, OpenDengue
Montserrat	PAHO, OpenDengue

Nepal	WHO/WPRO, EDCD, OpenDengue
New Caledonia	WHO/WPRO, DOH, OpenDengue
Nicaragua	PAHO, OpenDengue
Panama	PAHO, OpenDengue
Paraguay	PAHO, OpenDengue
Peru	PAHO, OpenDengue
Philippines	WHO/WPRO, OpenDengue
Puerto Rico	PAHO, OpenDengue
Saint Barthelemy	PAHO, OpenDengue
Saint Kitts and Nevis	PAHO, OpenDengue
Saint Lucia	PAHO, OpenDengue
Saint Martin	PAHO, OpenDengue
Saint Vincent and the Grenadines	PAHO, OpenDengue
Singapore	WHO/WPRO, MOH, OpenDengue
Suriname	PAHO, OpenDengue
Thailand	BoE, OpenDengue
Trinidad and Tobago	PAHO, OpenDengue
Turks and Caicos Islands	PAHO, OpenDengue
United States of America	PAHO, OpenDengue
Uruguay	PAHO, OpenDengue
Venezuela	PAHO, OpenDengue
Viet Nam	WHO/WPRO, OpenDengue
Virgin Islands (UK)	PAHO, OpenDengue
Virgin Islands (US)	PAHO, OpenDengue

Supplementary Table 6 Multicollinearity analysis for our regression model (DLM) based on the Variance Inflation Factor (VIF).

**Variable**	**VIF**	**Variable**	**VIF**	**Variable**	**VIF**
$\boldsymbol{NINO3.4_t}$	6.03	$deNINO3.4_t * \tau_i^{temp}$	6.78	$deNINO3.4_t * \tau_i^{precip}$	1.46
$\boldsymbol{NINO3.4_{t-1}}$	5.39	$deNINO3.4_{t-1} * \tau_i^{temp}$	6.05	$deNINO3.4_{t-1} * \tau_i^{precip}$	1.31
$\boldsymbol{NINO3.4_{t-2}}$	5.99	$deNINO3.4_{t-2} * \tau_i^{temp}$	6.73	$deNINO3.4_{t-2} * \tau_i^{precip}$	1.46

VIF values below 10 indicate low correlations between NINO3.4-derived variables in our model.

Supplementary Table 7 Bayesian estimates for our regression model.

**Variable**	**Estimate**	**Lower 95% CI**	**Upper 95% CI**
$\boldsymbol{deNINO3.4_t * \tau_i^{temp}}$	1.46	1.12	1.81
$\boldsymbol{deNINO3.4_{t-1} * \tau_i^{temp}}$	0.98	0.64	1.31
$\boldsymbol{deNINO3.4_{t-2} * \tau_i^{temp}}$	1.42	1.07	1.76
$\boldsymbol{deNINO3.4_t * \tau_i^{precip}}$	0.39	0.13	0.65
$\boldsymbol{deNINO3.4_{t-1} * \tau_i^{precip}}$	0.25	-0.01	0.50
$\boldsymbol{deNINO3.4_{t-2} * \tau_i^{precip}}$	0.53	0.26	0.79

Supplementary Text

Methods to estimate annual case counts for 2024 accounting for reporting delays and yet-to-be-reported data

Data

Dengue case data for the years 2023 and 2024 were obtained from the WHO Global Dengue Surveillance dashboard (hereafter, WHO dashboard) as of the reporting date of 1st March 2025. Additional data sources were searched and supplemented as needed, including the Pan American Health Organization (PAHO) Health Information Platform for the Americas Database (PLISA), WHO dengue situation updates for Western Pacific region, Ministry of Health annual bulletins, and government-operated news services. Due to reporting delays, Anguilla, Bhutan, Dominica, and Venezuela were yet to finalise their case counts for 2024 by 1st March 2025. We thus used a simple proportion of annual total cases by month model to predict their final 2024 annual case counts. Monthly total cases (newly reported suspected and confirmed cases) by country were used for analysis. Detailed sources of data for each country are available in Supplementary Table 5.

To project 2024 annual dengue cases for Anguilla, Bhutan, Dominica, and Venezuela, we leveraged historical dengue case data (1993-2023) for each country to calculate the proportion of cases observed by each month of the year. This allowed us to estimate the number of cases expected by the end of 2024 based on the trend from earlier months. Given the variability in available years by country, we first retrieved all accessible historical data for each country and assessed data coverage. Years with more than one month or four consecutive weeks of missing data were excluded, along with any years reporting all zero dengue cases.

Modelling

Corrections to account for delays in reporting

Evaluating stability of dengue cases reported on the WHO dashboard. The WHO dashboard receives updates at irregular intervals which have routinely scraped and stored in the following public GitHub repository (https://github.com/ahyoung-lim/DengueCrawler/tree/02eaee62f0c4d257e4a1e6d85ecc002a9da935ab). By considering the percent change of the monthly reported cases between updates, we assessed the stability of the reported data and the necessary adjustments for backfilling using a reporting factor.

Estimating the reporting factor. Using monthly reported cases, the reporting factor for each country at each lag in data reporting could be estimated using the following equation:

$$f_{c,d} = \frac{\frac{1}{T}\sum_{t=i}^{T}\left(\frac{N_{t,c,d}}{V_{t,c}}\right)}{1}$$

where N is the number of dengue cases for a given country (c), at a given epidemiological week (t) for a given delay (d), V is the validated count of dengue cases for a given country (c), at a given epidemiological week (t) and f is the reporting factor for a given country (c), at a given epidemiological week (t) for a given delay (d).

Estimation of 2024 annual counts

Counts reported at a weekly frequency were aggregated to return a monthly sum, facilitating their combination with monthly data for the same country. The cumulative sum of dengue case counts over each month was calculated annually for each country. The proportion of cumulative counts observed in each month of the year was then calculated by dividing cumulative counts for each month by the cumulative total for the whole year. The mean and standard deviation of this cumulative proportion across different years were calculated for each country. The mean cumulative proportion was used to extrapolate 2024 annual case load, based on the available 2024 counts (post adjustment for backfilling) and their date of last report. The standard deviation of the 2024 prediction was based on the standard deviation of the mean cumulative proportion across previous years for the relevant country.
