# Supplementary material for: Rising dengue risk with increasing El Niño–Southern Oscillation amplitude and teleconnections
Source: Nat Commun. 2025 Sep 29;16:8629. doi: 10.1038/s41467-025-63655-0 (PMC12480917; doi:10.1038/s41467-025-63655-0)
Supplement: Supplementary file 3 — Reporting Summary [file 41467_2025_63655_MOESM3_ESM.pdf]

nature portfolio

Corresponding author(s): Huaiyu Tian, Oliver J. Brady

Last updated by author(s): Jul 8, 2025

Reporting Summary

Nature Portfolio wishes to improve the reproducibility of the work that we publish. This form provides structure for consistency and transparency in reporting. For further information on Nature Portfolio policies, see our Editorial Policies and the Editorial Policy Checklist.

Statistics

For all statistical analyses, confirm that the following items are present in the figure legend, table legend, main text, or Methods section.

n/a	Confirmed	
☐	☒	The exact sample size (*n*) for each experimental group/condition, given as a discrete number and unit of measurement
☐	☒	A statement on whether measurements were taken from distinct samples or whether the same sample was measured repeatedly
☐	☒	The statistical test(s) used AND whether they are one- or two-sided<br>*Only common tests should be described solely by name; describe more complex techniques in the Methods section.*
☐	☒	A description of all covariates tested
☐	☒	A description of any assumptions or corrections, such as tests of normality and adjustment for multiple comparisons
☐	☒	A full description of the statistical parameters including central tendency (e.g. means) or other basic estimates (e.g. regression coefficient) AND variation (e.g. standard deviation) or associated estimates of uncertainty (e.g. confidence intervals)
☐	☒	For null hypothesis testing, the test statistic (e.g. *F*, *t*, *r*) with confidence intervals, effect sizes, degrees of freedom and *P* value noted<br>*Give P values as exact values whenever suitable.*
☐	☒	For Bayesian analysis, information on the choice of priors and Markov chain Monte Carlo settings
☐	☒	For hierarchical and complex designs, identification of the appropriate level for tests and full reporting of outcomes
☐	☒	Estimates of effect sizes (e.g. Cohen's *d*, Pearson's *r*), indicating how they were calculated

Our web collection on statistics for biologists contains articles on many of the points above.

Software and code

Policy information about availability of computer code

Data collection	The data used in this study were collected from open sources. The dengue case data are deposited in the OpenDengue database [https://opendengue.org/]. The climate index data are available from Climate index dataset of NCC [http://cmdp.ncc-cma.net/Monitoring/cn_index_130.php] and El Niño Index Dashboard of NOAA [https://psl.noaa.gov/enso/dashboard.html]. Local temperature and precipitation data are available from ERA5 [https://www.ecmwf.int/en/forecasts/datasets/reanalysis- datasets/era5]. The population data are available from GPW version 4 [https://sedac.ciesin.columbia.edu/data/collection/gpw-v4] and World Population Prospects 2024 [https://population.un.org/wpp/Download/Standard/Population/]. The CMIP6 ensembles are openly available via PCMDI/LLNL (California) [https://pcmdi.llnl.gov/CMIP6/].
Data analysis	The data analyses were performed in Python (3.9.19) and R (4.4.1). Code files to generate all the figures in the article are available on the following GitHub repository: https://github.com/huaiyutian/Dengue_ENSO.

For manuscripts utilizing custom algorithms or software that are central to the research but not yet described in published literature, software must be made available to editors and reviewers. We strongly encourage code deposition in a community repository (e.g. GitHub). See the Nature Portfolio guidelines for submitting code & software for further information.

Data

Policy information about availability of data

All manuscripts must include a data availability statement. This statement should provide the following information, where applicable:

- Accession codes, unique identifiers, or web links for publicly available datasets
- A description of any restrictions on data availability
- For clinical datasets or third party data, please ensure that the statement adheres to our policy

The dengue case data are deposited in the OpenDengue database [https://opendengue.org/]. The climate index data are available from Climate index dataset of NCC [http://cmdp.ncc-cma.net/Monitoring/cn_index_130.php] and El Niño Index Dashboard of NOAA [https://psl.noaa.gov/enso/dashboard.html]. Local temperature and precipitation data are available from ERA5 [https://www.ecmwf.int/en/forecasts/datasets/reanalysis- datasets/era5]. The population data are available from GPW version 4 [https://sedac.ciesin.columbia.edu/data/collection/gpw-v4] and World Population Prospects 2024 [https://population.un.org/wpp/Download/Standard/Population/]. The CMIP6 ensembles are openly available via PCMDI/LLNL (California) [https://pcmdi.llnl.gov/CMIP6/]. Source data are provided with this paper on the following GitHub repository: https://github.com/huaiyutian/Dengue_ENSO.

Research involving human participants, their data, or biological material

Policy information about studies with human participants or human data. See also policy information about sex, gender (identity/presentation), and sexual orientation and race, ethnicity and racism.

Reporting on sex and gender	This information has not been collected.
Reporting on race, ethnicity, or other socially relevant groupings	This information has not been collected.
Population characteristics	This information has not been collected.
Recruitment	This study involved no participants.
Ethics oversight	This study involved no participants.

Note that full information on the approval of the study protocol must also be provided in the manuscript.

Field-specific reporting

Please select the one below that is the best fit for your research. If you are not sure, read the appropriate sections before making your selection.

☐ Life sciences ☐ Behavioural & social sciences ☒ Ecological, evolutionary & environmental sciences

For a reference copy of the document with all sections, see nature.com/documents/nr-reporting-summary-flat.pdf

Ecological, evolutionary & environmental sciences study design

All studies must disclose on these points even when the disclosure is negative.

Study description	By incorporating reported dengue cases and climate data from 57 countries across the Americas and Asia from 1980 to 2024, we quantified the impact of global climate on dengue cases through local climate by defining country-level teleconnections between ENSO indices and population-weighted temperature and precipitation, with 2,280 observations analyzed using a distributed lag regression model.
Research sample	This study analyzed dengue cases reported from 57 tropical countries across the Americas and Asia and publicly-sourced global gridded climate data from 1980 to 2024.
Sampling strategy	We assembled the global dengue dataset from the OpenDengue website and other public sources, including the World Health Organization (WHO), the Pan-American Health Organization (PAHO), online databases, authorized Ministry of Health websites, our previously established dataset, and published literature.
Data collection	We conducted an extensive search for reported dengue cases and cross-verified data across various open sources. Data from these sources were aggregated from weekly, monthly, and quarterly intervals to construct yearly datasets. Y.X., Y.L., Z.Z., K.M.S., Y.C., K.J., A.C., A.L., and Y.W. collected the statistical data through public available sources online.
Timing and spatial scale	The dengue case data encompassed national-level number of annual reported cases as of December 31, 2024. Local climate observations were obtained from ERA5 monthly mean reanalysis product with a resolution of 0.25° × 0.25°, including air temperature at 2 meters above the Earth's surface and precipitation from 1980 to 2024. The Gridded Population of the World (GPWv4) data at the 15 arc-minute resolution was utilized to calculate the population-weighted climate for each country.
Data exclusions	Data from 2020 to 2024 were not included in the main model to avoid the impact of COVID-19.

Reproducibility	Reproducibility is ensured through openly accessible data sources and publicly available code supporting this study. All attempts to repeat the experiment were successful.
Randomization	Countries were categorized by continent for regional comparisons, ensuring proportional representation of all 57 tropical nations in the analytical groups.
Blinding	Blinding was not applicable as the data used in this study were derived from public sources, containing no personally identifiable information and requiring no participant involvement.

Did the study involve field work? ☐ Yes ☒ No

Reporting for specific materials, systems and methods

We require information from authors about some types of materials, experimental systems and methods used in many studies. Here, indicate whether each material, system or method listed is relevant to your study. If you are not sure if a list item applies to your research, read the appropriate section before selecting a response.

Materials & experimental systems

n/a	Involved in the study
☒	☐ Antibodies
☒	☐ Eukaryotic cell lines
☒	☐ Palaeontology and archaeology
☒	☐ Animals and other organisms
☒	☐ Clinical data
☒	☐ Dual use research of concern
☒	☐ Plants

Methods

n/a	Involved in the study
☒	☐ ChIP-seq
☒	☐ Flow cytometry
☒	☐ MRI-based neuroimaging

Plants

Seed stocks	N/A
Novel plant genotypes	N/A
Authentication	N/A
